# Solid pseudopapillary tumor of the pancreas: a rare entity in children

**DOI:** 10.11604/pamj.2020.35.137.22404

**Published:** 2020-04-27

**Authors:** Ghita Berrada, Soukaina Belaaroussi, Kamilia Chbani, Siham Salam, Dalal Laoudiyi, Lahcen Ouzidane, Asmaa El Kebir, Nisrine Bennani Guebessi, Samira Benayad, Farida Mernissi, Mehdi Karkouri, Salma Anis, Mounia Al Zemmouri

**Affiliations:** 1 Department of Pediatric Radiology, Abderrahim Harouchi Pediatric Hospital, Ibn Rochd University Hospital, Casablanca, Morocco; 2 Department of Pathology, Ibn Rochd University Hospital, Casablanca, Morocco; 3 Department of Surgery, Ibn Rochd University Hospital, Casablanca, Morocco

**Keywords:** Pseudopapillar, tumor, children, pancreas

## Abstract

Solid pseudopapillary tumors (SPTs) constitute 0.2 to 2.7% of non-endocrine primary tumors of the pancreas and comprise the majority (70%) of pediatric pancreatic neoplasms. These tumors are of unclear pathogenesis, low malignancy and favorable prognosis. Surgical resection offers an excellent chance for longterm survival, even in the presence of distant metastasis. The objective of this study is to review our experience in the management of SPT in a 12 years old girl at the pediatric hospital of the University hospital of Casablanca, in Morocco and provide an update on current management in pediatric population.

## Introduction

Solid pseudopapillary tumors (SPTs) are rare neoplasms accounting for 2% to 3% of pancreatic tumors and 0.9% to 2.7% of exocrine pancreatic neoplasms [1]. The first case of SPTs was reported by Virginia Frantz in 1959 [2]. This neoplasm, which has had a variety of names, was designated as SPT by the World Health Organization in 1996 [2]. SPTs primarily affect young females between 20 and 30 years. It is the most frequent pancreatic tumor in the second decade of life [3]. The majority of SPTs are indolent with favorable prognosis and have an excellent long-term outcome after complete surgical resection [3-5]. However, SPTs are considered malignant neoplasms due to metastases which are present at the time of diagnosis in 5% to 20% of the cases.

## Patient and observation

**Figure 1:**
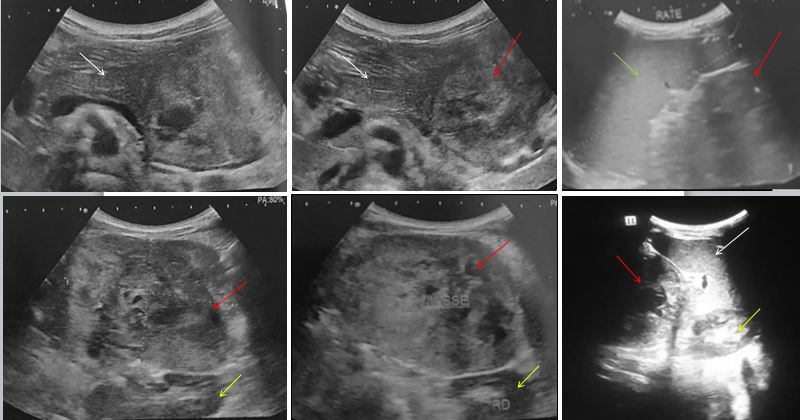
Ultrasound revealed a subcostal solid mass located on the left hypochondrium (red arrow), having a contact with the tail of the pancreas (white arrow) and the spleen (green arrow). It was hypoechoic, heterogenous. The left kidney was pushed back without being involved (yellow arrow)

**Figure 2:**
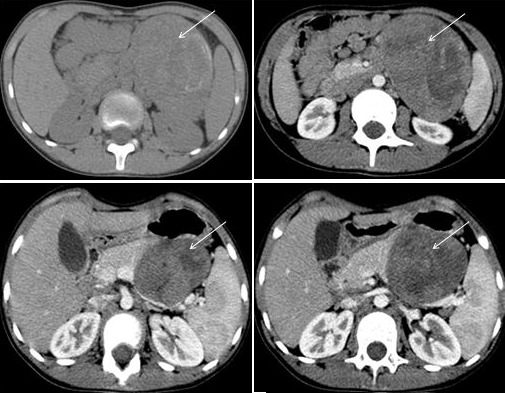
Computed tomography (CT) imaging identified a well-circumscribed mass located in the body and the tail of the pancreas (arrow). It was heterogeneous, containing cystic and solid portions, with peripheral calcifications. The solid portion had heterogenous enhancement after intravenous contrast administration, demonstrating some necrotic areas

We present our experience in the management of SPT in a 12 years old girl with no past medical history. The story of her illness began 2 months previously with abdominal pain without other associated signs. Abdominal examination revealed a palpable mass in the left hypochondrium. Ultrasound demonstrated a heterogeneous mass, having a contact with the tail of the pancreas and the spleen (Figure 1). On computed tomography (CT), this mass was located in the body and the tail of the pancreas. It was heterogeneous, solido-cystic, with peripheral calcifications, measuring approximatively 7 x 8 x 10.5 cm in maximum transverse, anterior-posterior and craniocaudal dimensions, respectively (Figure 2). Several lung nodules were present, considered as metastatic lesions. No other metastatic lesions were observed. As for serum tumor markers, all were negative: lactate deshydrogenase, α-fetoprotein, carbohydrate antigen 19-9 and uric acid. To determine the histological type of this tumor, we performed a percutaneous biopsy with ultrasound guidance.

**Figure 3:**
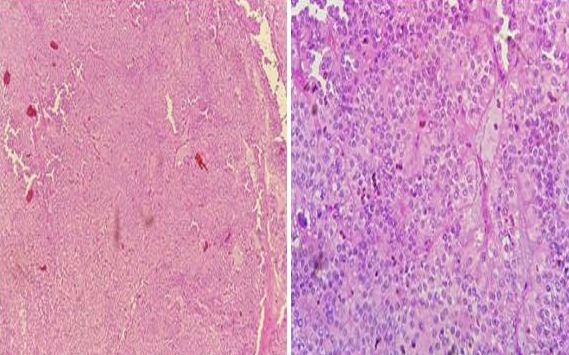
Pathologic results revealed a tumoral proliferation of rounded cells with a myxoid background. The architecture described layers of monomorphic cells with hyperchromatic nuclei and moderately abundant cytoplasm

**Figure 4:**
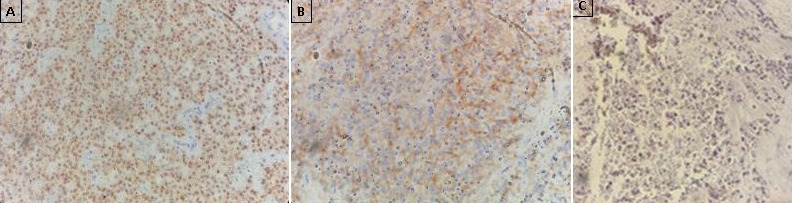
The tumor expressed the β-catenin [A] and CD56 [B]. CD99 which is a particular dot-like intracytoplasmatic marker, was highly expressed [C]

**Figure 5:**
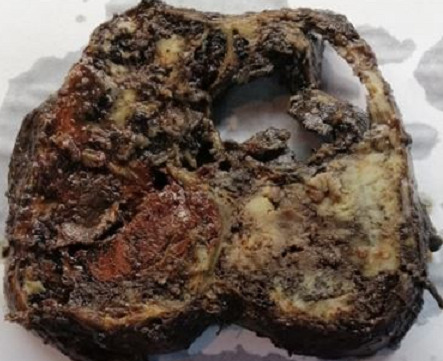
Gross appearance shows mixed solid whitish brown areas with foci of necrosis and hemorrhage and cystic areas containing necrotic fragments

Pathologic results, combined with immunohistochemical study were consistent with the diagnosis of SPT (Figure 3, Table 1). CD 99 which is a particular dot-like intracytoplasmatic marker, was highly expressed (Figure 4). This last appears to be highly unique for SPTs. Chromogranin and desmine markers were negative, thereby eliminating an endocrine tumor. The decision of the multi-disciplinary meeting was surgery without preoperative chemotherapy or radiotherapy. The tumor was resected en bloc with a corporo-caudal pancreatectomy without splenectomy. Macroscopically, it demonstrated characteristic areas of hemorrhage and necrosis, that were friable, with tissular portions having a brownish color (Figure 5). Histological examination showed a proliferation of tumors arranged in islets, layers, papillae, cords and tubes (Figure 3). Mitotic activity (Ki-67) was very low (5%). No perineural or vascular invasion was found. Surgical margins were negative. No postoperative diabetes was developed. Follow-up imaging will include CT at 3 to 6 month intervals and later annually.

**Table 1:** Immunohistochemistry for our case of SPT

		Marker
Positive staining	β-catenin	Wnt signaling
	Cytokeratin	Epithelium, ductal differentiation
	CD 56	
	CD 99	
Negative staining	Chromogranin	Neuroendocrine
	Desmine	Neuroendocrine
	Ki-67	Proliferation
Not performed	α-fetoprotein	Pancreatoblastoma
	α-1-antitrypsin	Exocrine acinar differentiation
	Vimentin	Mesenchyme
	CD10	Neuroendocrine
	Synaptophysin	Neuroendocrine

## Discussion

**Epidemiology:** SPTs comprise the majority of pediatric pancreatic neoplasms [5]. It is a unique tumor of low malignant potential, most commonly affecting non Caucasian (especially Asian and African) young women, between 20 and 40 years old [6,7]. The female to male ratio is 8-9 to 1 [3]. About 20-25% of the cases are seen in pediatric patients [8]. The origin and histogenesis of SPTs is still unknown, although it is thought to origi¬nate from totipotential stem cells with the capacity for both endocrine and exocrine differentiation [7].

**Clinical features:** Lee *et al.* demonstrated that SPTs in children presented with a palpable mass (60%), followed by abdominal pain (33.3%) [1]. SPTs are also often texturally fairly soft; accounting for the rarity of ductal dilatation and jaundice even for tumors arising in the pancreatic head [7].

**Biologic features:** pancreatic enzymes and common serum tumor markers (alpha-fetoprotein, carcinoembryonic antigen and beta-human chorionic gonadotrophin) are consistently negative, as was seen in our patient [3,5]. There are no known SPT-specific serum markers [3].

**Imaging features:** SPTs may appear in any part of the pancreas [1]. Tumor location in children is: tail (35.9% to 44%); head (30% to 34%); body and tail (10.3% to 13%) [1]. SPTs are usually large, well circumscribed and have both solid and cystic components [5]. Ultrasonographically, SPTs may appear as hypoechoic solid masses, solid masses containing cystic areas or cystic mass [4]. The fibrous capsule may be visualized as an echogenic or less commonly, hypoechoic rim [2]. The most helpful examination is the CT [9]. It helps to configurate the mass, determine the size, define the pancreatic anatomy and recognize the invasion into surrounding structures [4,10]. CT demonstrates a well circumscribed mass, surrounded by a fibrous capsule that may contain calcifications [2]. A delayed enhancement of the thick fibrous capsule can also be seen. SPTs may have variable composition: homogeneous solid, mixed and heterogeneous with juxtaposition of peripheric solid and central cystic zones; or cystic with thick wall [2]. Enhancement with intravenous contrast material is limited to solid portions, with unenhancing fluid and debris centrally [2].

Fluid-debris levels are seen in up to 20% of tumors. Less than one-third demonstrate internal septations. Duct dilatation is uncommon even with large masses in the head of the pancreas because SPTs often compress adjacent structures rather than invading them [2]. MRI is also useful; it provides information about resectability of SPTs [10]. Solid portions are iso- to hypointense to pancreas on T1-weighted images and slightly hyperintense to pancreas on T2-weighted images. A surrounding hypointense fibrous capsule and internal hemorrhage necrosis or debris, seen as high signal intensity on T1-weighted images, are distinguishing features of SPTs [2]. Lee *et al.* reported that solid pseudopapillary carcinoma may be differentiated from benign SPTs by its aggressive behavior, such as pancreatic duct dilation and vessel invasion, either with or without metastases. If such imaging features are present, aggressive surgical approach is mandatory and intensive follow-up is recommended [11]. Preoperative cytologic diagnosis can be confirmed by endoscopic ultrasound scan with fine-needle aspiration biopsy or transabdominal percutaneous biopsy using a trocar needle, with ultrasound or CT-guidance but present a risk of tumor dissemination, so his indication remains controversial [3,8,9].

**Pathology:** SPTs are a rare epithelial solid tumor of the pancreas that invariably develop significant cystic degeneration, acquiring the characteristic solid-cystic appearance.

**Gross appearance:** macroscopically, SPTs are usually large, well circumscribed and have mixed solid whitish brown areas with foci of necrosis and hemorrhage and cystic areas containing necrotic fragments. As they increase in size, the areas of cystic-hemorrhagic degeneration grow until they get simular to a pseudocyst [3,9].

**Histologic appearance:** histologically, SPTs have a very characteristic appearance of solid areas composed with of poorly cohesive cells that form pseudopapillae around thin blood vessels. These polygonal epithelioid cells have medium-size, soft appearance, basophilic cytoplasms, large rounded or ovoid nuclei, with reinforced nuclear membrane and thin chromatin [3,9]. Foaming histiocytes, cholesterol crystals, fibrosis and calcifications can be found. Very rare mitosis are observed; glycogen or mucin are not observed. The tumor cells are PAS and Alcian blue negative [9]. The histological features that lead to aggressive behavior, malignant potential and the worst prognosis are vascular, neural invasion and involvement of the adjacent pancreas, adding in some reports the degree of nuclear atypia, high mitotic index and many apoptotic features. If these criteria are presented, some authors suggest calling them “solid pseudopapillary carcinomas” [9].

**Immunohistochemical:** immunohistochemical study is very useful, revealing a diffuse positivity for certain histochemical markers like neuron-specific enolase (NSE), vimentin, CD-10 and béta-catenin, which is not specific [9]. α1-antitrypsin and α1-antichymotrypsin are intensely positive, but in a small group of cells [9]. Immunostaining for estrogen receptors, especially progesterone receptors, is sometimes positive; this marking evokes a possible hormonosensitivity of SPTs and could explain the female predominance [6]. Notohara and coworkers found that SPTs exhibited unique immunohistochemical features with expression of CD56, CD10 and local expression of other neuroendocrine markers. These findings suggest that papillary and solid epithelial cells are with predominance of exocrine features but having the capacity for dual (exocrine and endocrine) differentiation [10]. Immunostaining for chromogranin is negative, thereby eliminating an endocrine tumor that is the main differential diagnosis [6]. Cells are consistently negative for mucin (ductal origin), enzymes (acinar origin) and hormones (endocrine origin), which supports the theory that SPTs arise from an embryonal pancreatic pluripotent cell [3]. According to recently published data, a particular dot-like intracytoplasmatic expression of CD99 is highly unique for SPTs [3]. To date, this cytoplasmatic paranuclear “dot-like” pattern has not been described in any other type of endocrine or exocrine pancreatic tumors included in the differential diagnosis of SPTs [3].

**Treatment and prognosis:** the current recommendations concur that children with pancreatic SPTs should undergo complete surgical resection that is dictated by tumor location and remains the treatment of choice [5]. Distal pancreatectomy with or without splenic preservation can be performed for tumors in the body or tail and pancreaticoduodenectomy (Whipple or Longmire procedure) for tumors of the head [4,9]. Invasion to the portal vein or superior mesenteric artery should not be included as a criterion for nonresectability. Extensive lymphatic dissection or more radical local approaches are not indicated [4]. Chemotherapy, radiation and hormone therapy are rarely used; their indication is discussed particularly in non-localized forms. Surgery is curative in more than 95% of localized tumors. Survival after complete excision of the tumor is 80-90%; the recurrence rate is 10 to 15%. Malignant forms account for about 15% of cases. The criteria for malignancy are determined by vascular, ganglionic, perineural invasion of neighboring organs and tumor size greater than 5cm. The prognosis of SPTs is favorable, even in the presence of metastases [10]. Metastases can be hepatic, peritoneal or pulmonary [6]. There is general consensus that surgical debulking (in contrast to other pancreatic malignancies) should be performed for these metastases [4]. Although surgical resection is generally curative, a close follow-up is advised in order to diagnose a local recurrence or distant metastasis and choose the proper therapeutic option for the patient [12].

**Differential diagnosis:** despite the technological advances, preoperative diagnosis is difficult because of the similarity of findings among other pancreatic tumors [4]. In children, secondary pancreatic involvement of tumors such as neuroblastoma, leukemia, lymphoma and lymphoproliferative disorders is more common than primary neoplasms like pancreatoblastoma [4,6]. The imaging features of SPTs reflect the pathologic findings of cystic and solid components, intratumoral hemorrhage, a fibrous capsule and less commonly, calcification. When present, the fibrous capsule and internal hemorrhage are the features that distinguish SPTs from other pancreatic tumors [2].

## Conclusion

SPTs constitute the majority (70%) of pediatric pancreatic neoplasms. It is a rare exocrine pancreatic tumor that typically have the form of a well-encapsulated mass with solid and cystic components due to varying degrees of internal hemorrhage and necrosis. These tumors are of unclear pathogenesis, low malignancy and surgical resection offers an excellent chance for longterm survival, even in the presence of distant metastasis. Although surgical resection is generally curative, a close follow-up is advised in order to diagnose a local recurrence or distant metastasis and choose the proper therapeutic option for the patient.

## Competing interests

The authors declare no competing interests.
